# 

**Published:** 1891-11

**Authors:** 


### IOWA STATE VETERINARY MEDICAL ASSOCIATION.

The fourth annual session of the Iowa State Veterinary Medical Association will be held in the parlors of the Savery House, Desmoines, Iowa, November 12th and 13th, 1891.

S. Stewart, *Secretary.*

---

### THE ILLINOIS STATE VETERINARY MEDICAL ASSOCIATION.

The annual meeting of the Illinois State Veterinary Medical Association will take place at the Sherman House, Chicago, on the 17th and 18th of November at 10 A. M.

The following papers will be read: Dr. D. McIntosh, Parturient Apoplexy in Cows; Dr. S. H. Kingery, Pleuro-pneumonia in Horses; Dr. A. H. Baker, Azoturia; Dr. R. W. Story, Pulsatilla, its Actions and Uses; Dr. Paul Paquin, The War of Cells; Dr. D. Wilson, Influenza; Dr. F. S. Schoenleber, Veterinary Agricultural Editorials; Dr. G. Z. Barnes, Tetanus; Dr. J. A. McDonnell, Glanders; Dr. O. J. Lanigan, Rare Cases; Dr. Khlotel, Tuberculosis; Dr. F. S. Billings, Recent Investigations; Dr. R. J. Withers, The Use of Antiseptics.

New members will be admitted and officers will be elected.

S. S. BAKER, *President*.

### BOOKS AND PAMPHLETS RECEIVED.

On the Comparative Osteology of the United States Columbibæ, by R. W. Shufelt, C. M. Z. S., Rep. from the Pro. Zoo. Society, London.

Journal and Proceedings, Royal Society, New SouthWales, Sidney, 1890.

Journal of the Asiatic Society of Bengal, Calcutta, 1891.

Verhandlungen des Naturhistorischen Vereines der preussichen Rhin. lands, Westfalens und des Reg. Bezirks Oznabrüeck, Bonn, 1891.

Sitzungsberichte der Naturforscher Gesselschaft bei der Universitat Dorpat, 1891.

Mittheilungen des NaturwissenschaftenVereins für Steirmark Gratz, 1891.

Verhandlungen und Mittheillungen des Siebenburgischen Vereins für Naturwissenschaften in Hermannstadt.

Jahrbericht des Vereins für Naturwissenschaft zu Braunschweig, 1891.

Jenaische Zeitschrift für Naturwissenschaft herausgegeben von der medizinisch naturwissenschaftliche Gesellschaft zu Jena, 1891.

Jahresbricht der Gesaellschaft für natur und Heilkunde in Dresden, 1891.

Vierteljahrschrift der Naturforschend Gesselschaft in Zurich, 1890-91.

Verhandlungen der Schweizerischen Naturforschenden Gesellschaft in Davos, 1891.

Compte Rendu des travaux presentés à la soixante-treizième session de la Societé Helvétique des Sciences Naturelles Davois, 1890.

